# Supplementary material for: Garlic-Induced Enhancement of Bifidobacterium: Enterotype-Specific Modulation of Gut Microbiota and Probiotic Populations
Source: Microorganisms. 2024 Sep 28;12(10):1971. doi: 10.3390/microorganisms12101971 (PMC11509698; doi:10.3390/microorganisms12101971)
Supplement: Supplementary file 1 [file microorganisms-12-01971-s001.zip › microorganisms-3225613-supplementary.pdf]

Garlic-Induced Enhancement of *Bifidobacterium*: Enterotype-Specific Modulation of Gut Microbiota and Probiotic Populations

Jina Ha[1,†], Jinwoo Kim[1,2,†], Seongok Kim[1,2], Kwang Jun Lee[3,*], and Hakdong Shin[1,2,*]

[1]Department of Food Science and Biotechnology, College of Life Science, Sejong University, Seoul 05006, Republic of Korea; jinaha423@korea.kr (J.H.); jinwoo3239@sejong.ac.kr (J.K.); skim01@sejong.ac.kr (S.K.); hshin@sejong.ac.kr (H.S.)

[2]Carbohydrate Bioproduct Research Center, College of Life Science, Sejong University, Seoul 05006, Republic of Korea

[3]Division of Zoonotic and Vector Borne Diseases Research, Center for Infectious Diseases Research, National Institute of Health, Cheongju 28159, Republic of Korea; kwangjun@korea.kr (K.J.L.)

*Correspondence to: hshin@sejong.ac.kr (H.S.) and kwangjun@korea.kr (K.J.L.)

†These authors contributed equally to this work.

Supplemental data

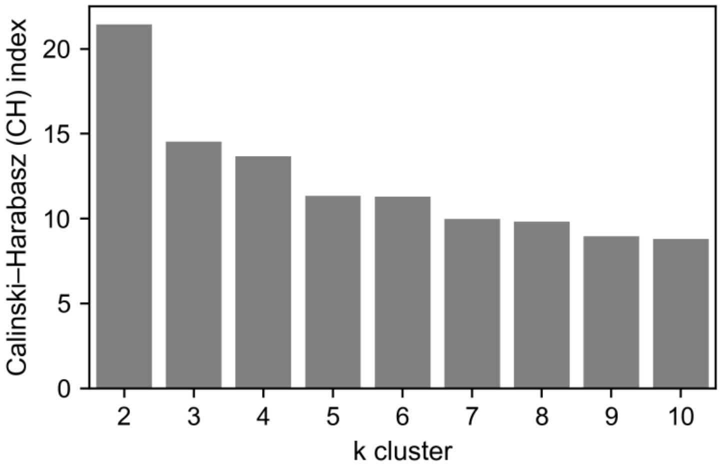

Figure S1. Calinski-Harabasz index for determining optimal cluster number. Calinski-Harabasz (CH) index values were calculated for different numbers of clusters (k) ranging from 2 to 10.

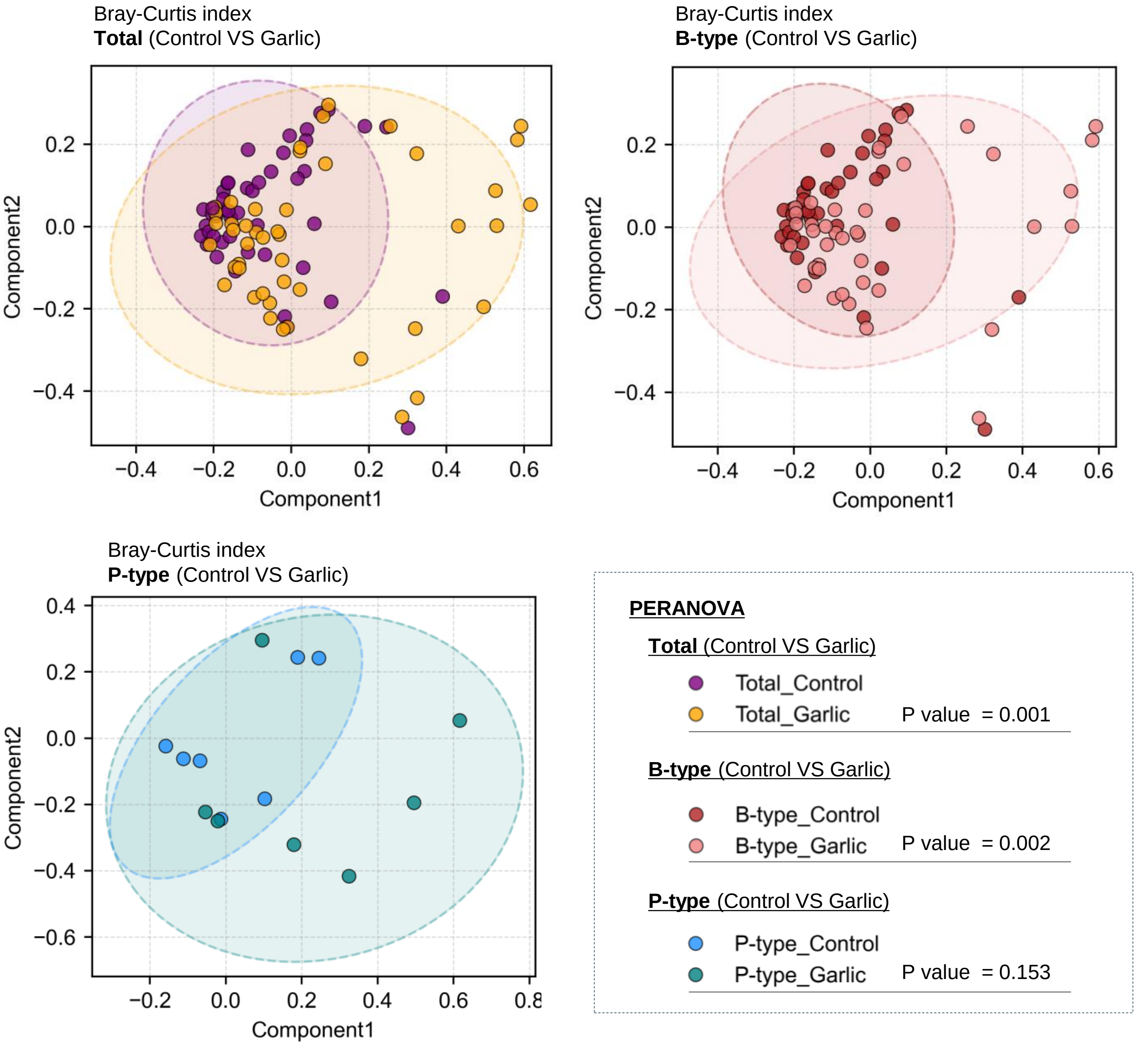

Figure S2. Beta diversity analysis of genus-level microbial communities in response to garlic. Principal Coordinates Analysis (PCoA) plots, based on the Bray-Curtis index calculated at the genus level, were used to assess the beta diversity of microbial communities between control and garlic treatment groups. The ellipses represent 90% confidence intervals around the group centroids. The PERMANOVA results on the right indicate significant differences in beta diversity between control and garlic groups.

Supplemental data

Table S1. The bacterial strains used in this study

Strains	Origin	Source
Bifidobacterium adolescentis	Human feces of a healthy, 27-year-old male, Korea	In this lab
Bifidobacterium faecale	Human feces of a two-week-old baby, Korea	In this lab
Bifidobacterium longum	Human feces	In this lab
Bifidobacterium suillum	Feces of piglets, Bologna, Italy	In this lab
Bifidobacterium pseudocatenulatum	Human feces, Korea	In this lab

Supplemental data

Table S2. Twelve ASV features mapped with the *Bifidobacterium*

Amplicon sequence variants	Genus-levels (SILVA DB)	BLAST search
0db5d53458bf68ab4f3ca669bff9b5f3	*Bifidobacterium*	*B. adolescentis, B. faecale*
7da3ceb01197526fe2bad855e06ed528	*Bifidobacterium*	*B. adolescentis, B. faecale*
91f820042ffc5e3161e13698e04d1918	*Bifidobacterium*	*B. adolescentis, B. faecale*
a984458fd71c5210bea51e0153f97698	*Bifidobacterium*	*B. adolescentis, B. faecale*
886934d21d1dc0553c7d9f0cf22c0832	*Bifidobacterium*	*B. animalis subsp*
99d97cd7fb7385dd6d697d0b516f58af	*Bifidobacterium*	*B. bifidum*
89ee04c35dae073d6f107a9e0133b97f	*Bifidobacterium*	*B. bifidum*
1dba26afd5b4b5c8c41147bb753dd538	*Bifidobacterium*	*B. longum*
b2b9d4f508cfe07e4b77ec6177d3e449	*Bifidobacterium*	*B. longum*
5f577142f0e35a8760df42bcdc5f00d1	*Bifidobacterium*	*B. pseudocatenulatum*
7151308deafd6db48a802cb9df76daaa	*Bifidobacterium*	*B. pseudocatenulatum*
8c1eb8c5183c1c1235c8b7d2136a3196	*Bifidobacterium*	*B. pseudocatenulatum*
